# Slow degrading Mg-based materials induce tumor cell dormancy on an osteosarcoma-fibroblast coculture model

**DOI:** 10.1016/j.bioactmat.2021.12.031

**Published:** 2021-12-30

**Authors:** Philipp Globig, Regine Willumeit-Römer, Fernanda Martini, Elisa Mazzoni, Bérengère J.C. Luthringer-Feyerabend

**Affiliations:** a Institute of Metallic Biomaterials, Helmholtz-Zentrum Hereon, 21502, Geesthacht, Germany; b Department of Medical Sciences, University of Ferrara, 44121, Ferrara, Italy

**Keywords:** Cancer, Magnesium degradation, Ki-67, Proliferation inhibition, pH, Alkalization

## Abstract

Osteosarcoma is one of the most common cancers in young adults and is commonly treated using surgery and chemotherapy. During the past years, these therapy approaches improved but failed to ameliorate the outcomes. Therefore, novel, targeted therapeutic approaches should be established to enhance treatment success while preserving patient's quality of life. Recent studies suggest the application of degradable magnesium (Mg) alloys as orthopedic implants bearing a potential antitumor activity. Here, we examined the influence of Mg-based materials on an osteosarcoma-fibroblast coculture. Both, Mg and Mg–6Ag did not lead to tumor cell apoptosis at low degradation rates. Instead, the Mg-based materials induced cellular dormancy in the cancer cells indicated by a lower number of Ki-67 positive cancer cells and a higher p38 expression. This dormancy-like state could be reversed by reseeding on non-degrading glass slides but could not be provoked by inhibition of the protein kinase R-like endoplasmic reticulum kinase. By investigating the influence of the disjunct surface-near effects of the Mg degradation on cell proliferation, an increased pH was found to be a main initiator of Mg degradation-dependent tumor cell proliferation inhibition.

## Highlights

• Mg-based material degradation induce cellular dormancy in osteosarcoma cells.
• Dormancy state is indicated by a lower expression of Ki-67 and higher expression of p38 compared to non-degrading materials.
• The alkalization during Mg material degradation is the driving force of cancer cell proliferation inhibition.

## Graphical abstract

**Figure:**
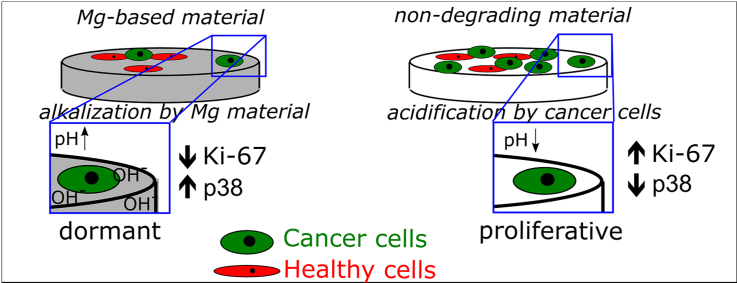

## 1 Introduction

Osteosarcoma (OS) accounts for approx. 20% of all primary bone tumors and is therefore the most common pediatric cancer type arising from bone forming cells [1]. Despite successful treatment approaches, OS is still responsible for around 10% (bones and joints) of cancer-related deaths in children [2]. Low grade OS is commonly treated by limb salvage (80–90% of patients), which includes surgical excision and a subsequent bone reconstruction [1]. Depending on the tumor region in the bone and the grading, neoadjuvant chemotherapy is applied, which failed to significantly increase the patients’ survival rates for the last 20 years [3]. A novel treatment approach may be the application of magnesium (Mg)-based materials, combining non-weight bearing bone replacements with a local cancer treatment. Mg-based materials are already employed for orthopedic applications due to their good biocompatibility, degradability and comparable mechanical properties to those of bones [4,5]. Mg degradation is characterized by the occurrence of surface-near effects, the most obvious being an elevation of pH, osmolality and Mg^2+^ (or other alloying elements) and a constant release of hydrogen gas (H_2_), which can be tailored by influencing the degradation rate [6]. Therefore, aside from the application as a bone replacement, the surface-near effects may have additional effects on residual tumor cells of the inaccessible bone regions. In fact, some recent studies suggested cytotoxic effects on tumor cells provoked by specific Mg alloy degradation [[7], [8], [9]]. By introducing alloying elements like silver, the degradation rate and biological responses of Mg-based materials can be tailored as desired. The aim is to adjust the degradation rate to specifically target tumor cells and preserving the integrity of surrounding healthy cells. Therefore, we recently described an optimized osteosarcoma-fibroblast coculture model to study the antitumoral activity of Mg-based materials using fluorescent cells as a monitoring system on opaque materials [10]. Consecutive investigations focused on the tumor cytotoxicity and proliferation under normoxic and hypoxic conditions, since tumors comprise hypoxic regions, where the oxygen level fall below 3% [[11], [12], [13], [14]]. H_2_ has been recently described as a possible cancer treatment approach due to its beneficial action on colorectal cancer prognosis and chemotherapy outcomes [15,16]. Additionally, the effect of H_2_ release from Mg-based materials on cancer cells was recently studied Zan et al. [17], which is why this publication focuses more on the other surface-near effects such as an increased pH, osmolality, and Mg concentration in the surrounding.

The Mg-based materials may also serve as base materials for drug delivery approaches. Therefore, the materials may be loaded with drugs, such as cytostatics, which are released from the degrading material and can directly target adjacent cancer cells in the implant area. For this porous material may be beneficial as shown by Qian and colleagues [18,19].

One of the tumor-specific characteristics is the limitless, rapid cell proliferation and therefore excessive tumor growth [20]. This acquired phenotype is mainly provoked by mutations leading to a constitutively activation of proteins that are involved in proliferation associated signaling cascades or inactivation of proliferation suppressors [21]. These include the common signaling pathways PI3K/Akt/mTOR, mitogen-activated protein (MAP) kinase and Notch signaling [22]. On the other hand, there are signaling pathways that take a role in tumorigenesis, e.g., tumor growth factor (TGF-)β signaling. TGF-β signaling is known to promote tumor cell invasion and metastases but suppresses tumor cell proliferation by stopping cell cycle progression and induction of G1-arrest [23]. However, tumor cells can evade this proliferation suppression by inactivating mutations of downstream proteins like SMAD or initiation of inhibitors of cyclin-dependent kinases (CDK) to keep the cell cycle running [24]. In theory, TGF-β signaling can be linked to Mg degradation through the cofactor activity of Mg^2+^ [25], while another proliferation-associated protein, interleukin-8 (IL-8), is secreted by long-lasting tumor cell exposure to hypoxia and decreased pH [26]. Moreover, IL-8 was already reported as a potential autocrine growth factor, which was associated with increased tumor cell proliferation [[27], [28], [29], [30], [31]].

In opposite to the limitless cell proliferation, cancer cells can also switch into dormancy, a process by which tumor cells stop cell cycle progression and subsequent proliferation in response to stressors (harsh environment conditions, therapeutics) to avoid cell damage and apoptosis [32]. Dormancy can be roughly divided into two forms: cellular dormancy and tumor mass dormancy [33]. During cellular dormancy, a certain amount of cells undergo G0-G1 cell cycle arrest and stop proliferating. Here, an increased expression of p38 is associated with tumor cell dormancy [34]. Tumor mass dormancy describes an equilibrium of proliferating and dying cells. In this form the cells are more active compared to cellular dormancy as tumor growth is physically limited. Stress induced dormancy is mainly induced via p38 signaling cascade [35] with subsequent activation of activating transcription factor 6α (ATF6α) and protein kinase RNA-activated (PKR)-like ER kinase (PERK). Both are responsible for the maintenance of the dormant phenotype and survival mechanisms [32].

With this study we aim to elucidate further the effects of Mg-based materials on osteosarcoma to promote their use as novel targeted therapy. We focused on deeper understanding of the influence of Mg and Mg–6Ag on cell cytotoxicity using an osteosarcoma-fibroblast coculture model. Moreover, we investigated the cell proliferation with the specific marker Ki-67 and potential influenced proliferation signaling pathways. Finally, we considered the Mg degradation dependent surface-near effects separately to further understand the observed influences of Mg and Mg–6Ag on cancer cells.

## 2 Materials and methods

### 2.1 Surface treatment and cleaning of Mg, Mg–6Ag and Ti–6Al–4V

Material surface treatment and cleaning procedure were conducted as previously described [10]. Pure Mg (99.95%) and Mg–6Ag (Mg with 6 wt % Ag) were fabricated by permanent mold gravity casting (Helmholtz-Zentrum Hereon, Geesthacht, Germany) and extrusion into rods (10 mm diameter). Subsequently, processed rods (9 mm diameter) were cut into disks (1.5 mm thickness; Henschel KG, Munich, Germany) and stored under argon atmosphere until use. Table 1 shows the chemical composition of the used Mg and Mg–6Ag material.

**Table 1:** Chemical composition of Mg and Mg–6Ag presented as weight %. Ag = silver; Al = aluminum; Cu = copper; Fe = iron; Mg = magnesium; Ni = nickel.

	Chemical composition in weight %					
	Mg	Ag	Fe	Cu	Ni	Al
**Mg**	99.94	<0.00005	0.0048–0.0049	0.0002–0.0003	<0.0002	0.013–0.016
**Mg–6Ag**	≈94	5.94–6.34	0.0019–0.0021	0.0013–0.0014	0.009–0.0010	<0.0100

To clear impurities on the material surface that affect Mg degradation, Mg and Mg–6Ag disks were ground (Saphir 360 from ATM GmbH, Mammelzen, Germany) with SiC 2500 grid paper (Starcke GmbH & Co.KG, Melle, Germany) at 80 rpm. Subsequent cleaning procedure was conducted in an ultrasonic bath (Branson 1210, Branson Ultrasonics, Danbury, USA): n-hexan, acetone, and ethanol (Merck KGaA, Darmstadt, Germany). Then, samples were sterilized in 70% ethanol and preincubated in 2 mL Dulbecco's modified eagle medium GlutaMAX-I (DMEM GlutaMAX-I; Life Technologies, Darmstadt, Germany) supplemented with 10% fetal bovine serum (FBS, Merck KGaA, Darmstadt, Germany) for 24 h. Ti–6Al–4V samples were used as a material control. Initially, Ti-based samples were cut from a round bar (F.W. Hempel Legierungsmetall GmbH and Co. KG, Oberhausen, Germany) and were polished. After every use, Ti–6Al–4V samples were ultrasonically cleaned in 2% Hellmanex II solution (Hellma Materials GmbH, Jena, Germany), chloroform, and 100% ethanol (Merck KGaA, Darmstadt, Germany), each for 20 min, and sterilized by autoclaving.

The mean degradation rate of the Mg-based materials was determined by weight loss measurement. The samples were initially weighed after the cleaning procedure. Following the immersion in cell culture medium (2 mL), the samples were treated with chromic acid (180 g/L in distilled water, VWR International, Darmstadt, Germany) to eliminate the residual degradation layer. For more details, see our previous work [10].

### 2.2 Cell culture

Saos-eGFP was kindly provided by Prof. Tognon (Department of Medical Sciences, University of Ferrara, Italy). This cell line originated from the human osteosarcoma cell line Saos-2 and was genetically modified by introducing the gene for the enhanced green fluorescent protein (eGFP). The detailed procedure is described by Morelli et al. [36]. RF fibroblasts were obtained from Innoprot (Innoprot, Derio, Spain). These cells are derived from primary human dermal fibroblasts and genetically manipulated to constitutively express the red fluorescent protein FP602. Both, tumor cells and fibroblasts were maintained in DMEM GlutaMAX-I supplemented with 10% FBS under cell culture conditions (37 °C, 5% CO_2_, 95% relative humidity (rH)).

### 2.3 Cell seeding on Mg, Mg–6Ag and Ti–6Al–4V

All experiments analyzing the effect of the aforementioned materials on the osteosarcoma-fibroblast coculture were executed in 24-well plates by directly seeding the cells on the material surface. Therefore, Saos-eGFP and RF Fibroblasts were detached by applying 0.05% trypsin-EDTA (Life Technologies GmbH, Darmstadt, Germany) and counted with a CASY Cell Counter (Roche Diagnostics GmbH, Mannheim, Germany). Cell suspensions were mixed to a 1:1 ratio of Saos-eGFP and RF Fibroblasts for the coculture with a total number of 10,000 cells. The optimal cell ratio and cell amount were determined in a previous study [10]. The amount of 10,000 cells in monocultures of both cell types served as controls. Cell suspensions containing the monocultures and the coculture were set to a volume of 40 μL and directly applied to the surface of preincubated Mg, Mg–6Ag, Ti–6Al–4V or glass slides. After allowing the cells to adhere to the surface for 20 min, 2 mL of cell culture medium were applied to each well and changed every 2–3 days. Cells were cultured on degrading material (Mg, Mg–6Ag) and non-degrading material (Ti control, glass control) for the indicated timespans under cell culture conditions in normoxia (20% O_2_) or hypoxia (3% O_2_) before analyzing.

To inhibit the proliferation of Saos-eGFP and RF Fibroblasts, the cells were starved in serum-free medium overnight and incubated with mitomycin c (MMC) prior to the seeding on the materials. No deleterious effect of MMC on cells such as cytotoxicity were detected (data not shown). Cells were kept in a T25 cell culture flask (Greiner Bio-One International GmbH, Kremsmünster, Austria) and washed once with phosphate buffered saline (PBS). Then, 10 μg/mL (Saos-eGFP) or 50 μg/mL MMC (RF Fibroblasts) in serum-free cell culture medium was added to the cells for 2 h. Afterwards the MMC solution was removed by three washing steps with PBS and the cells were counted for cell seeding on the materials.

### 2.4 Determination of cell numbers on materials

After one, three and seven days, microscopic images of cell-seeded Mg, Mg–6Ag and Ti–6Al–4V (Ti control) were taken with an upright fluorescence microscope (Eclipse Ni-E; Nikon GmbH, Dusseldorf, Germany). Likewise, images of glass slides were taken using an inverted fluorescence microscope (Eclipse Ti–S; Nikon GmbH, Dusseldorf, Germany). Cell number counting was conducted with ImageJ (Rasband, W.S., ImageJ, U.S. National Institutes of Health, Bethesda, Maryland, USA, https://imagej.nih.gov/ij/, 1997–2018) as previously described [10]. Cell numbers of the monocultures or both cell types in the coculture were counted by thresholding and analyzing particles.

### 2.5 Determination of tumor cell cytotoxicity

To assess the cytotoxic potential of the Mg-based materials, three different approaches, (Ii) lactate dehydrogenase (LDH) assay (based on plasma membrane integrity), (ii) “Vivafix Cell Viability Assay” (relying on primary amines cellular location), and (ii) caspase-3 and reactive oxygen species (ROS) staining, were selected.

LDH was quantified in the cell culture supernatant using the “Cytotoxicity Detection KitPLUS LDH” kit (Roche, Basel, Switzerland). Supernatants of samples and controls at day 7 were transferred to a 96-well plate (50 μL). The Reaction mixture, consisting of the catalyst and dye solution, was prepared as suggested by the manufacturer and 50 μL were added to each well. After incubating for 30 min in the dark, the absorbance was read at 490 nm using a microtiter plate reader (Sunrise™ Tecan microplate reader; Tecan, Männedorf, Switzerland). The resulting absorbance values obtained from three samples in triplicates of Mg, Mg–6Ag, Ti–6Al–4V and a lysed control were averaged and set in relation to the glass control.

Vivafix Cell Viability Assay was used to assess viability by flow cytometry. After seven days, cells were detached from the material by 0.05% trypsin-EDTA (Life Technologies GmbH, Darmstadt, Germany) for 5 min and centrifuged for 5 min at 200×*g* (Rotina 420; Andreas Hettich GmbH & Co. KG, Tuttlingen, Germany). Cells were then washed in PBS and resuspended in 500 μL PBS in flow cytometry tubes (Becton Dickinson, Franklin Lakes, NJ, USA). Afterwards, 1 μL of VivaFix 547/573 was added to each sample, mixed, and incubated for 30 min at room temperature in the dark. Cells were again washed twice in PBS, resuspended in 500 μL PBS and analyzed with an S3e cell sorter (Biorad, Hercules, CA, USA) with the laser configuration 488, 561 nm.

Furthermore, caspase-3 substrates and ROS were visualized to analyze tumor cell apoptosis upon seeding on Mg-based materials. Seven days after cell seeding, supernatants were replaced by a mix of 2 μM NucView 405 substrate (Biotium Inc., Hayward, CA, USA) and 5 μM of CellROX Deep Red reagent (Thermo Fisher Scientific, Waltham, MA, USA) in cell culture medium and incubated for 30 min at 37 °C. Afterwards, images of the living cells were taken with a confocal laser scanning microscope (DM 6000 CS, Leica, Wetzlar, Germany) using the following laser wavelengths: 405 nm (caspase-3), 488 nm (Saos-eGFP), 552 nm (RF Fibroblasts) and 633 nm (ROS). The resulting images of ROS staining were quantified with ImageJ with subsequent calculation of the corrected total cell fluorescence (CTCF) of outlined cells (CTFC=Integrated density of cells - cell area X average mean gray value of the background) in the ROS channel image (2–3 cells per image). For caspase-3 staining, the total image field of view was outlined to subsequently calculate the corrected total field fluorescence (CTFF) with the help of three background positions (CTCF=Integrated density of total field – field area x average mean gray value of the background). Both calculations aim to eliminate background dependent biased results.

### 2.6 Immunofluorescence staining of Ki-67 and p38

All antibody dilutions were performed in PBS supplemented with 10% FBS. FBS was used to keep Mg samples passivated and ensure staining quality. Three and seven days after seeding, cells were fixed on the material with 2% paraformaldehyde (Alfa Aesar, Haverhill, MA, USA) in PBS supplemented with 10% FBS for 20 min at room temperature. Afterwards, cells were permeabilized using 0.5% Triton X-100 (Thermo Fisher Scientific, Waltham, MA, USA) for further 20 min at room temperature. To screen proliferating tumor cells, anti-Ki-67-PerCP-Vio700, Clone REA183 (Miltenyi Biotec, Bergisch Gladbach, Germany) was diluted 1:50 and mixed with 5 μg/mL 4′,6-Diamidin-2-phenylindol (DAPI, Sigma-Aldrich Chemie GmbH, Munich, Germany) in PBS supplemented with 10% FBS and added on the material surface for 20 min at room temperature in the dark. Afterwards, cells were washed three times with PBS supplemented with 10% FBS. For PERK inhibition, the selective inhibitor GSK2606414 (Hölzel Diagnostika, Cologne, Germany) was added to the 2 mL cell culture medium to a final concentration of 5 μM for 72 h. Afterwards, Ki-67 was stained as described above. Images were taken with a confocal laser scanning microscope (cLSM) (DM 6000 CS, Leica, Wetzlar, Germany) using the following laser wavelengths: 355 nm (DAPI), 488 nm (Saos-eGFP, Ki-67) and 552 nm (RF Fibroblasts).

For p38 visualization, permeabilized cells were initially blocked in PBS with 10% FBS for 30 min. Afterwards the cells were washed three times with PBS supplemented with 10% FBS. Then, 50 μL of 5 μg/mL primary mouse p38 alpha MAPK14 antibody (Santa Cruz Biotechnology Inc., Dallas, TX, USA) were added for 60 min at RT and cells were washed again three times. Afterwards, cells were incubated with the 5 μg/mL secondary goat anti-mouse IgG (H + L) antibody conjugated with CF®405 M (Biotium Inc., Hayward, CA, USA) for additional 60 min in the dark. The cells were washed again three times and visualized. Images were taken with a confocal laser scanning microscope (DM 6000 CS, Leica, Wetzlar, Germany) using the following laser wavelengths: 355 nm (p38), 488 nm (Saos-eGFP) and 552 nm (RF Fibroblasts). The p38 expression was quantified by calculating the CTCF of three cells from three images.

### 2.7 Enzyme-linked immunosorbent assay of IL-8 and SMAD2/SMAD3

With the enzyme-linked immunosorbent assay (ELISA) the concentrations of IL-8 and the activated, phosphorylated SMAD2/SMAD3 (Phospho SMAD2/SMAD3) complex in the supernatant of the coculture on Mg, Mg–6Ag, Ti–6Al–4V and glass slides were quantified. Human IL-8 DuoSet ELISA (R&D Systems, Minneapolis, MN, USA) was performed as described by the manufacturer. In brief, capture antibody was diluted in PBS to 4 μg/mL and incubated in 96 well plates (High binding, Sarstedt AG & Co., Nürnbrecht, Germany) over night at room temperature. After three washing steps with wash buffer (0.05% Tween 20 in PBS) using a wash station (Bioplex Pro Wash station, Biorad, Hercules, CA, USA) the plates were blocked using reagent diluent (1% bovine serum albumin, BSA in PBS) for 1 h at room temperature. Following another washing step, 100 μL of standard or supernatant diluted in reagent diluent if necessary was added and incubated for 2 h at room temperature. This followed again a washing step and the addition of the detection antibody diluted in reagent diluent (20 ng/mL) for 2 h at room temperature and a washing step. Then, horse radish peroxidase (HRP) conjugated on streptavidin was added to each well and incubated for 20 min in the dark followed by an additional washing step. Finally, substrate solution was added for 20 min and the resulting color reaction (blue) was stopped by adding H_2_SO_4_ and the color change (yellow) was measured with a plate reader (Sunrise™ Tecan microplate reader; Tecan, Männedorf, Switzerland) set to 450 nm. IL-8 concentrations were calculated from the absorbance values with the help of a standard curve in triplicates for three samples of one condition. Concentrations were normalized to the cell number per sample.

Relative quantities of phosphorylated SMAD2/SMAD3 complex were measured using the PathScan Sandwich Elisa Kit (Cell Signaling Technology, Danvers, MA, USA). Cell-seeded materials were rinsed once in ultrapure H_2_O and cells were lysed with the implied lysis buffer as suggested by the manufacturer. Cell lysates were applied to the microwells and incubated for 2 h at 37 °C followed by a washing step. Then, the detection antibody (1 h), HRP-linked secondary antibody (30 min) and substrate solution (10 min) were added and incubated at 37 °C for the indicated timespans. A washing step was applied between the single incubation steps. Finally, a stop solution as added and the absorbance was measured with a plate reader (Sunrise™ Tecan microplate reader; Tecan, Männedorf, Switzerland) set to 450 nm. Resulting absorbance values (duplicates of two samples) were normalized to the cell number.

### 2.8 Adjusting pH, osmolality and Mg concentration

To track the selected surface-near effects of the complex Mg degradation, the medium pH, osmolality, and Mg concentration were studied and adjusted individually.

For pH adjustment, the CO_2_ concentration in the incubator (BBD 6220, Thermo Electron LED GmbH, Langenselbold, Germany) was changed to 0.7% and the resulting pH of the medium was measured after 2 h with an ion-sensitive field-effect transistor (ISFET) pH sensor (Sentron SI600, Sentron Europe BV, Roden, The Netherlands) and compared to the control (5% CO_2_).

Osmolality was increased using polyethylene glycol (PEG) 400. PEG 400 was diluted in cell culture medium to a concentration of 60 mM. The resulting osmolality was measured using the freezing point osmometer ‘Osmomat Auto’ (Gonotec GmbH, Berlin, Germany) and compared to the control (cell culture medium).

To increase Mg concentration in the cell culture medium, a Mg extract was produced according to EN ISO standard 10993–12:2012. Mg cubes (0.2 g/mL) were immersed in DMEM supplemented with 10% FBS for 72 h. Then, the resulting extract was sterile filtered (Thermo Fisher Scientific, Waltham, MA, USA) and Mg concentration was quantified by atomic absorption spectrophotometer (AAS), as previously described [10]. The Mg^2+^ concentration in the undiluted extract was 30 mM and was diluted 1:6 to achieve 5 mM (average Mg^2+^ concentration during Mg-based material degradation according to previous findings [10]).

The influence of the selected Mg degradation-dependent surface-near effects on the expression of the proliferation marker Ki-67 was determined as described in chapter 2.6. Both, Saos-eGFP and RF Fibroblasts were seeded on glass slides in 24-well plates in a ratio of 1:1 with a total number of 10,000 cells. Then, the manipulated cell culture media with increased pH (0.7% CO_2_), osmolality (PEG 400) or Mg concentration (30 mM and 5 mM) were applied to the coculture and Ki-67 expression was analyzed after seven days.

### 2.9 Statistical analysis

The data of the cell numbers on the different materials were obtained from three independent experiments with three samples each. Image acquisition and quantification of the proliferation marker Ki-67 was conducted for all conditions from one experiment with two samples and three randomly chosen positions. Data is always shown as the arithmetic mean ± standard deviation (m±sd), if not stated otherwise. Statistical analysis (Prism 6, GraphPad Software, La Jolla, CA, USA) was conducted as follows: Comparison of (1) reseeded samples and control with an unpaired non-parametric *t*-test (Mann–Whitney test), (2) cells on different material or different time points using non-parametric one-way analysis of variances (ANOVA) (Kruskal–Wallis test) with Dunn's multiple comparison test, or (3) cells on different materials at different time points using a two-way ANOVA with Tukey's multiple comparison test.

## 3 Results

### 3.1 Progression of tumor cell and healthy cell growth on Mg-based materials

**Fig. 1:**
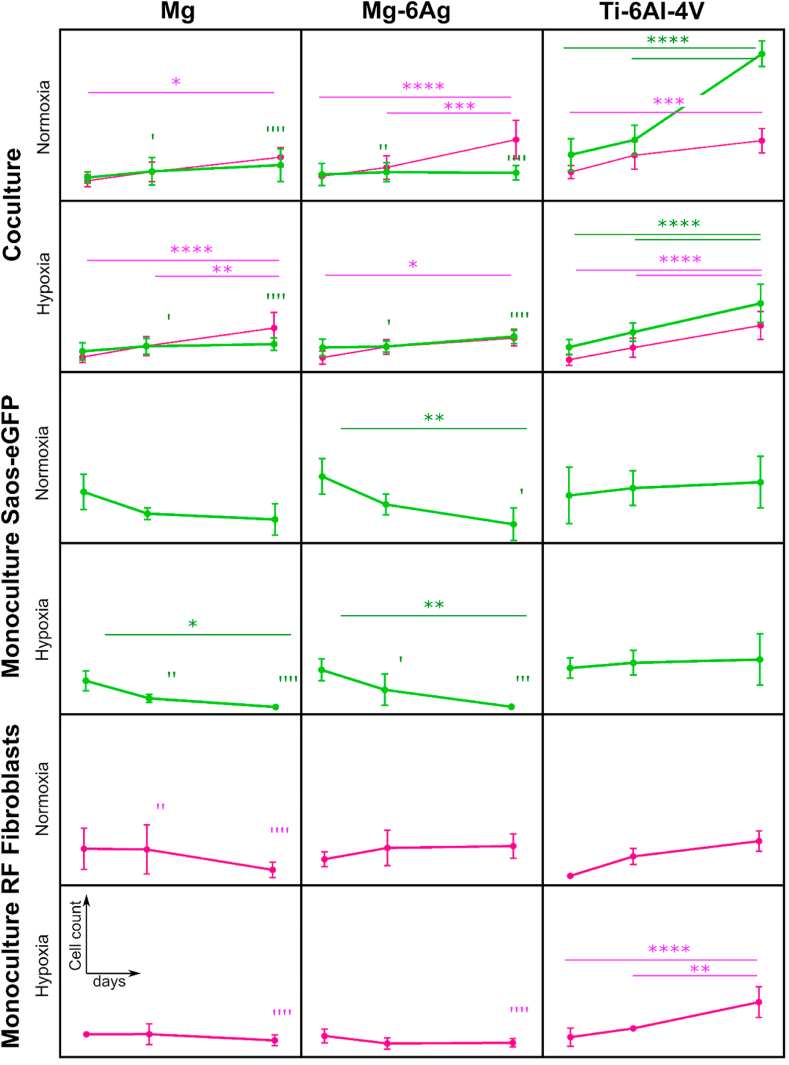
Impact of Mg, Mg–6Ag and Ti–6Al–4V on tumor cell and healthy cell numbers under hypoxia and normoxia. Saos-eGFP (green) and RF Fibroblasts (magenta) were seeded in 1:1 coculture or as monocultures on Mg, Mg–6Ag and Ti–6Al–4V. Cell numbers are shown after one, three and seven days as the arithmetic m±sd of three independent experiments with three samples per time point. Significant differences between different time points (*) or to the cell number on Ti–6Al–4V as a non-degrading control (’) were obtained via a two-way ANOVA with Tukey's multiple comparison test (n = 9); one symbol = p < 0.05; two symbols = p < 0.01; three symbols = p < 0.001; four symbols = p < 0.0001. (For interpretation of the references to color in this figure legend, the reader is referred to the Web version of this article.)

The degradation rates and concentrations of Mg and Ag after immersion of the here used slow-degrading Mg-based materials are shown in (Table 2). For details, see our previous work [10]. A degradation rate of 0.2 mm/a after seven days of immersion was determined for these Mg and Mg–6Ag materials. Tumor cells and healthy fibroblasts were seeded on Mg, Mg–6Ag and non-degrading Ti–6Al–4V in coculture or monoculture (Fig. 1).

**Table 2:** Mean degradation rates of Mg and Mg–6Ag presented as the arithmetic m±sd (n = 6). nd = not determined.

Time of immersion (days)	Mg			Mg–6Ag		
	MDR (mm/a)	c_Mg_ (mM)	c_Ag_ (mM)	MDR (mm/a)	c_Mg_ (mM)	c_Ag_ (μM)
1	0.73 ± 0.25	2.46 ± 0.52	nd	0.63 ± 0.31	1.25 ± 0.36	0.25 ± 0.20
3	0.25 ± 0.02	3.75 ± 0.83	nd	0.29 ± 0.06	2.13 ± 0.31	0.17 ± 0.08
7	0.20 ± 0.06	4.02 ± 0.48	nd	0.18 ± 0.07	2.54 ± 0.74	0.24 ± 0.10

In coculture, tumor cell numbers remained comparatively constant on the here used degrading Mg and Mg–6Ag materials, while rising on the non-degrading Ti–6Al–4V, under normoxia and hypoxia. Therefore, the number of cancer cells on Mg and Mg–6Ag was already significantly lower compared to that on Ti–6Al–4V after three days. In contrast to this, healthy cell numbers steadily and significantly increased within the observation time of seven days irrespective of degrading or non-degrading material. Therefore, the healthy cell numbers did not differ significantly on the different materials on the respective time points. Cell numbers of Saos-eGFP in monoculture seemed to significantly decrease on Mg and Mg–6Ag, while slightly increasing on non-degrading materials. RF Fibroblast cell numbers remained comparatively constant on degrading material, whereas on Ti–6Al–4V a slight elevation was observed. The relative cell numbers of the coculture were previously shown [10].

### 3.2 Assessing cytotoxic potential of slow degrading extruded Mg and Mg–6Ag

A constant tumor cell number might indicate a scenario of balanced tumor cell death and proliferation. Therefore, the slow degrading Mg and Mg–6Ag were tested for their cytotoxic potential with different tests. The release of lactate dehydrogenase (LDH) during cell growth on the materials revealed that there were no significant differences in the absorbance values of Mg, Mg–6Ag, Ti–6Al–4V and the glass control (Table 3, left column). Though, the LDH release of lysed cells was significantly higher compared to the degrading materials and the glass control indicating cell integrity on these materials. Analyzing cytotoxicity via flow cytometry showed similar findings (Table 3, right column). For this test, a dye was applied that bind to primary amine groups on the cell membrane on intact cells. In dead cells this dye can permeate the cell and binds to intracellular amines, giving a higher amount of fluorescence. These flow cytometry results showed again no significant differences in the plots of degrading and non-degrading materials. The majority of the cells exhibited only few fluorescence signals, indicating intact cells.

**Table 3:** Tumor cytotoxicity of Mg-based materials. Supernatant LDH and dead cell numbers obtained from flow cytometry are presented as the arithmetical m±sd relative to the glass control. Statistics: Kruskal-Wallis H test with Dunn's multiple comparison test (n = 6); ** = p < 0.01, **** = p < 0.0001.

	relative supernatant LDH	relative dead cell numbers
glass control	1.00	1.00
lysis control	4.70 ± 0.50****	18.43 ± 5.52**
Mg	1.05 ± 0.17	2.60 ± 3.64
Mg–6Ag	1.10 ± 0.18	1.79 ± 1.74
Ti–6Al–4V	1.16 ± 0.05	3.79 ± 3.02

**Fig. 2:**
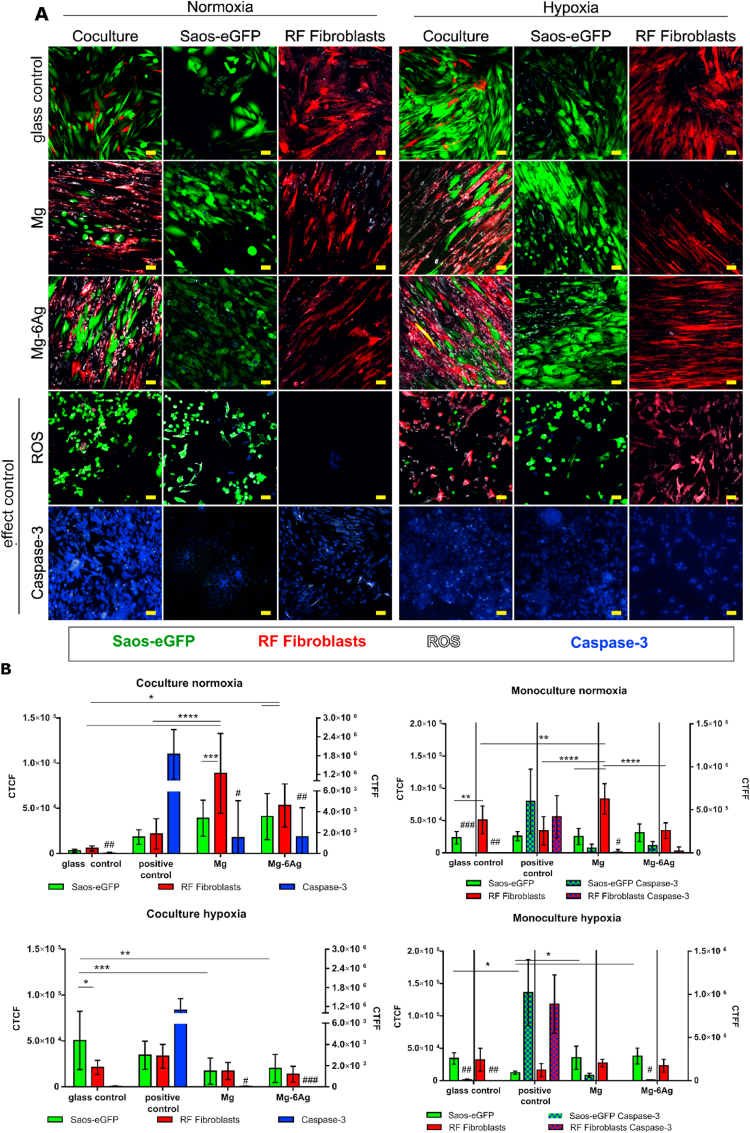
Influence of Mg-based materials on ROS production and caspase-3 activation. (A) cLSM: Saos-eGFP (green), RF Fibroblasts (red), ROS (white), caspase-3 (blue). Conditions: coculture, monoculture, 7 days. Effect controls: Caspase-3^+^(atmospheric conditions), ROS^+^ (100 μM menadione). Scale bar 50 μm. (B) Quantification: Calculation of the corrected total cell fluorescence (CTCF) (ROS) of two cells at three positions of two distinct samples (n = 12). CTCF values: Saos-eGFP (green), RF Fibroblasts (red) as the arithmetical m±sd. Calculation of the corrected total field fluorescence (CTFF) (caspase-3) of three positions of two distinct samples (n = 6). CTFF values: caspase-3 (blue) as m±sd. Significance differences between CTCF were obtained via a two-way ANOVA (cell type, material) with Tukey's multiple comparison test (n = 9); * = p < 0.05; ** = p < 0.01; *** = p < 0.001; **** = p < 0.0001. Significance differences between CTFF of the Caspase-3 control, Mg and Mg–6Ag to the glass control were obtained via a Kruskal-Wallis H test with Dunn's multiple comparison test; # = p < 0.05; ## = p < 0.01; ### = p < 0.001. (For interpretation of the references to color in this figure legend, the reader is referred to the Web version of this article.)

To confirm the results that the cells majorly remained intact upon seeding on Mg materials, the production of ROS and activity of caspase-3 that introduce apoptosis were visualized. Fig. 2 displays the images obtained by the cLSM showing Saos-eGFP (green) and RF Fibroblasts (red) in coculture or monocultures. The oxidative stress (white) and caspase-3 activity (blue) are indicated for both cell types grown on Mg and Mg–6Ag under normoxia and hypoxia. Quantification of these images revealed that oxidative stress in coculture cells under normoxia was significantly higher on Mg and Mg–6Ag materials compared to the glass control. The results for the monocultures under normoxia were comparable, except that the oxidative stress of the cells in monocultures on glass tended to be higher compared to the coculture. The production of ROS was diminished on the Mg and Mg–6Ag materials under hypoxic conditions compared to respective samples under normoxia. The quantification of caspase-3 activity revealed no significant differences between cells grown on degrading and non-degrading materials independent of mono- or coculture and oxygen-level.

### 3.3 Mg and Mg–6Ag induce a reversible tumor cell dormancy-like phenotype

**Fig. 3:**
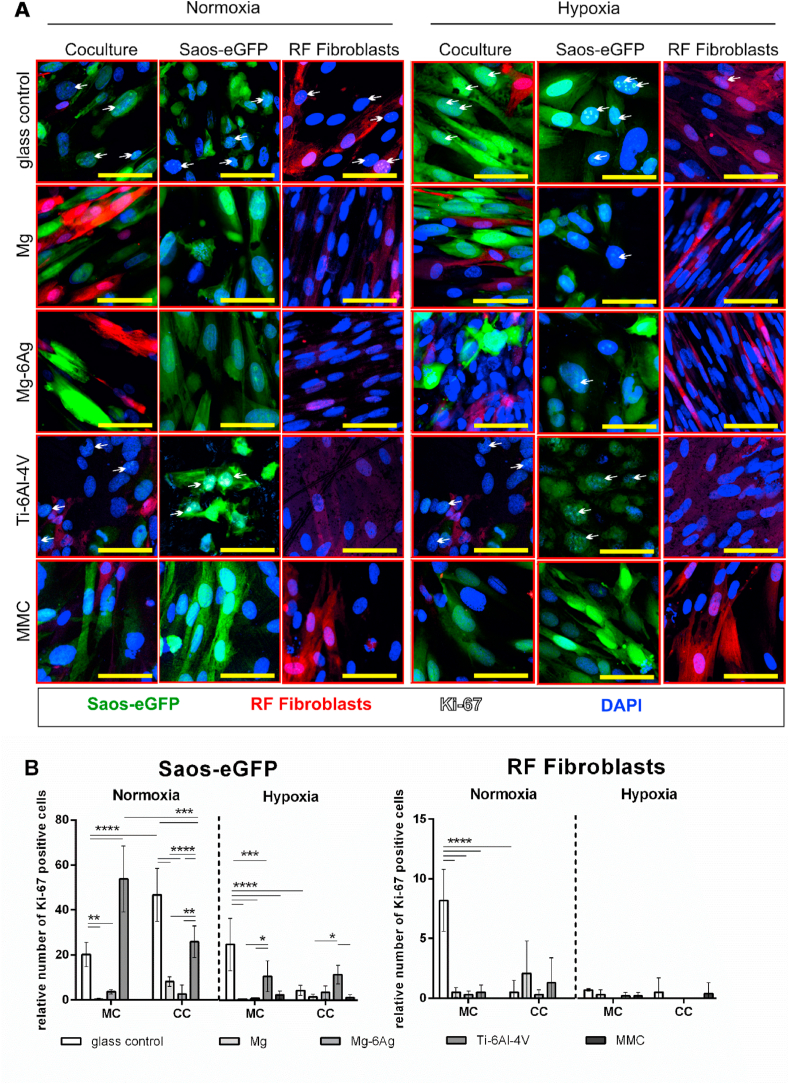
Impact of Mg-based materials on cell proliferation, by Ki-67 immunostaining. (A) cLSM: Saos-eGFP (green), RF Fibroblasts (red), Ki-67 (white indicated by arrows), nuclei DAPI (blue). Conditions: normoxia, hypoxia, 7 days. Scale bar 50 μm. (B) Quantification: relative Ki-67 positive tumor (Saos-eGFP) and healthy cells (RF Fibroblasts): m±sd from two samples with three randomly chosen positions. Significance differences between relative Ki-67 positive cells on different materials and under different conditions were obtained via a two-way ANOVA with Tukey's multiple comparison test (n = 6); * = p < 0.05; ** = p > 0.01; *** = p < 0.001; **** = p < 0.0001. (For interpretation of the references to color in this figure legend, the reader is referred to the Web version of this article.)

**Fig. 4:**
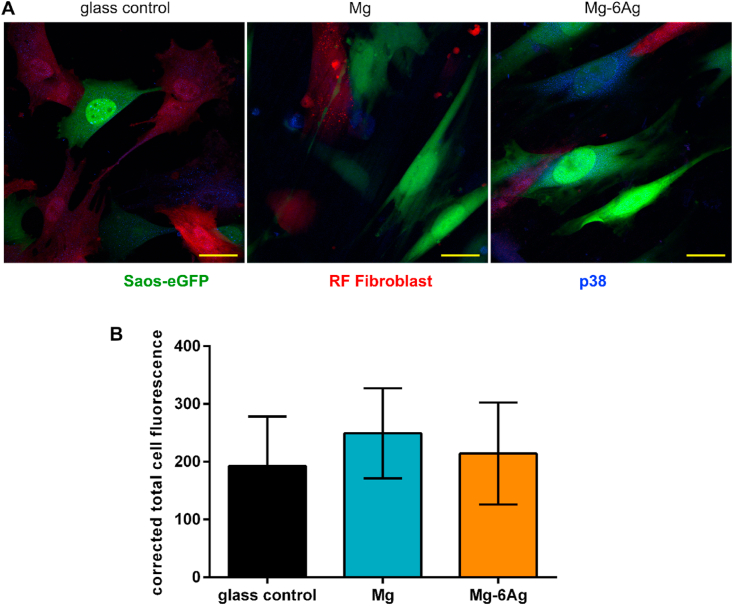
The impact of Mg-based materials on p38 expression in tumor cells. (A) Representative images of p38 (blue) expression in Saos-eGFP (green) and RF Fibroblasts (red) in coculture. Scale bar is 30 μm. (B) Quantification of p38 in Saos-eGFP by calculating the CTCF of three cells/image from three images/material (n = 9). Statistics: Kruskal-Wallis H test with Dunn's multiple comparison test. (For interpretation of the references to color in this figure legend, the reader is referred to the Web version of this article.)

Taken together, Table 3 and Fig. 2 suggested no cytotoxic potential of the here used slowly degrading Mg and Mg–6Ag materials. Another explanation for the constant numbers of tumor cells within the coculture observed in Fig. 1 involves a diminished cell proliferation. To examine this, tumor cells and fibroblasts were seeded on degrading and non-degrading materials and Ki-67 expression was visualized after seven days. Ki-67 is a proliferation marker, and its expression elevates during the progression of the cell cycle. Fig. 3A visualizes the expression of Ki-67 in Saos-eGFP and RF Fibroblasts in mono- and coculture on Mg, Mg–6Ag, Ti–6Al–4V and the glass control under normoxia and hypoxia. Fig. 3B quantifies the staining and shows the relative numbers of Ki-67 positive tumor cells and healthy cells. Overall, Ki-67 expression tended to be higher under normoxia compared to hypoxia. Moreover, the relative number of proliferating tumor cells was significantly higher on non-degrading material (Ti–6Al–4V, glass control) compared to degrading material (Mg, Mg–6Ag). Furthermore, cells that were proliferation inhibited by MMC treatment showed a decreased Ki-67 expression as expected. In contrast to this, proliferation of healthy cells was not affected, which indicated that Mg and Mg–6Ag specifically inhibited tumor cell proliferation and forced the cells into a dormancy-like state. To investigate whether this cancer cell proliferation inhibition is explainable by a dormancy-like state, the expression of p38 in Saos-eGFP was examined (Fig. 4). Both degrading materials, Mg and Mg–6Ag, tended to increase the p38 expression in the cancer cells compared to the glass control. This supported the hypothesis that the cancer cells are forced into a dormancy-like phenotype.

This dormancy-like phenotype of the osteosarcoma cells was shown to be reversible. Tumor cells and fibroblasts were grown in coculture on Mg and Mg–6Ag. As shown, this led to few proliferating cells. Detachment and reseeding of the cells on glass slides significantly increased the relative number of Ki-67 positive tumor cells to around 55% (Table 4).

**Table 4:** Reversibility of the tumor cell dormancy-like phenotype. Cells were seeded on Mg and Mg–6Ag, detached and reseed on glass slides. Relative Ki-67 positive cells are shown as m±sd from two samples with three randomly chosen positions for each sample. Significance differences between relative Ki-67 positive cells on Mg or Mg–6Ag or their reseeded pairs were obtained via a Mann-Whitney test (n = 6); ** = p < 0001.

	relative Ki-67 positive cells	
	Saos-eGFP	RF Fibroblasts
Mg	8.2 ± 2.1	2.1 ± 2.7
reseeded from Mg	55.4 ± 10.5**	0.0 ± 0.0
Mg–6Ag	2.7 ± 3.9	0.3 ± 0.4
reseeded from Mg–6Ag	55.2 ± 21.4**	0.0 ± 0.0

### 3.4 Tumor cell proliferation inhibition signaling pathway

**Fig. 5:**
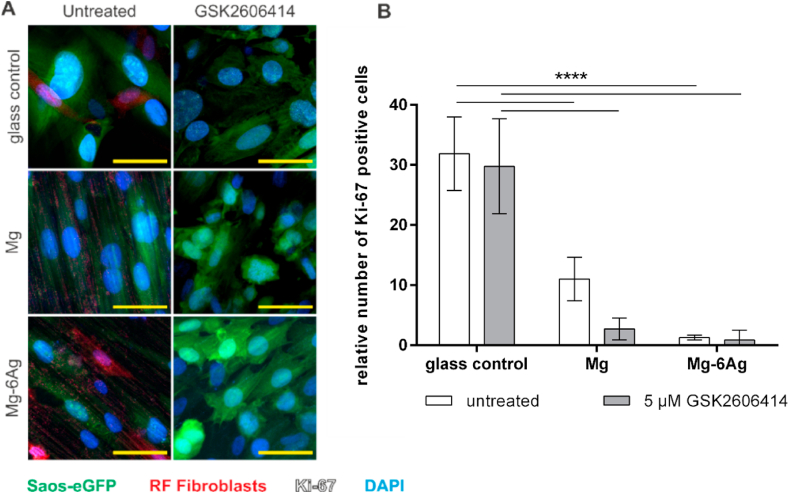
Influence of PERK inhibitor GSK2606414 on tumor cell proliferation on Mg, Mg–6Ag. (A) cLSM: Saos-eGFP (green), RF Fibroblasts (red, Ki-67 (white), cell nuclei (blue). Conditions: normoxia, 5 μM GSK2606414 for 72 h. Glass control without PERK inhibitor treatment. Scale bar 50 μm. (B) Quantification: relative numbers of Ki-67 positive cells (coculture) as m±sd from two samples with three randomly chosen positions for each sample. Significance differences between relative Ki-67 positive cells on different materials and treatment were obtained via a two-way ANOVA with Tukey's multiple comparison test (n = 6); **** = p < 0.0001. (For interpretation of the references to color in this figure legend, the reader is referred to the Web version of this article.)

To investigate the dormancy-like phenotype of the tumor cells on a molecular level, two pathways were studied: The PERK pathway and TGF-β signaling pathway. Upon unfavorable environmental conditions or stress induction, PERK is able to induce a cell cycle arrest. Inhibiting PERK might reverse this cell cycle arrest induction. Fig. 5A presents the Ki-67 expression in tumor and healthy cells on degrading material with addition of the PERK inhibitor GSK2606414. Though, this PERK inhibition did not appear to significantly increase relative numbers of Ki-67 positive tumor cells on the here used Mg and Mg–6Ag materials as shown in Fig. 5B.

**Fig. 6:**
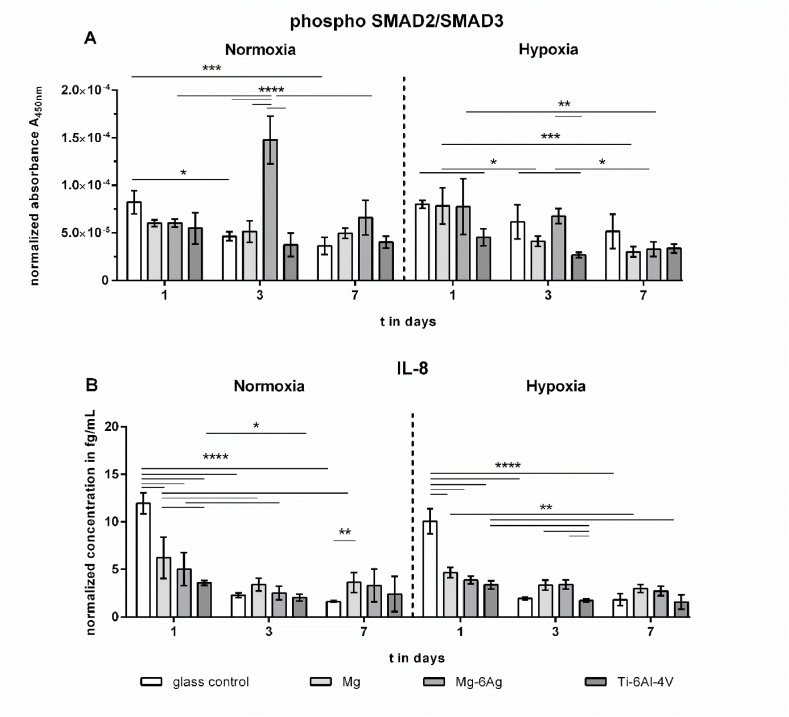
Phosphorylated SMAD2/SMAD3 and IL-8 in the supernatant. Concentrations are normalized to the cell number. Resulting protein concentrations are shown as m±sd from triplicates of two experiments with three samples. Statistics: protein concentrations on different materials and time points were obtained via a two-way ANOVA with a Tukey's multiple comparison test; one symbol = p < 0.05; two symbols = p < 0.01; four symbols = p < 0.0001.

The TGF-β signaling pathway is also able to regulate cell proliferation by suppressing cell cycle progression through cyclin-dependent kinase (CDK) inhibitors. Fig. 6 shows the normalized concentrations of TGF-β signaling associated proteins: Phosphorylated SMAD/SMAD3 complex in the cell lysate and Interleukin 8 (IL-8) in the supernatant of the coculture seeded on degrading and non-degrading material. Both under normoxia and hypoxia, the normalized concentrations of phosphorylated SMAD2/SMAD3 complex did not significantly differ between degrading and non-degrading material. IL-8 secretion can be an event of hypoxia or low environmental pH. Therefore, IL-8 concentrations in the supernatants of the coculture on Mg, Mg–6Ag, Ti–6Al–4V and the glass control were quantified. Fig. 6B shows that the normalized IL-8 concentrations after seeding on glass tended to be initially higher compared to Mg and Mg–6Ag materials.

### 3.5 Initiation of proliferation inhibition by selected effects of Mg degradation

**Fig. 7:**
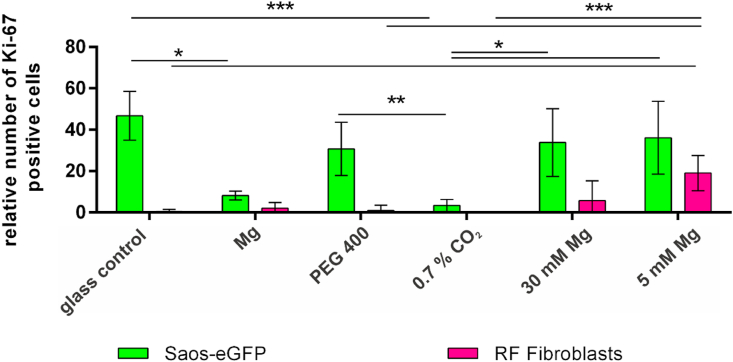
Impact of selected parameters of Mg degradation on cell proliferation Ki-67 positive cells were counted and set in relation to total tumor cells (green) and total healthy cells (magenta). Relative Ki-67 positive cells are shown as m±sd from two samples with three randomly chosen positions for each sample. Samples were coculture seeded on Mg or glass slides (glass control) with 60 mM polyethylene glycol 400 (PEG 400), 0.7% CO_2_, or Mg extracts with indicated Mg concentrations. Statistics: relative Ki-67 positive cells (Saos-eGFP or RF Fibroblasts) under different conditions were obtained via a Kruskal-Wallis H test with Dunn's multiple comparison test (n = 6); * = p < 0.05; ** = p < 0.01; *** = p < 0.001. (For interpretation of the references to color in this figure legend, the reader is referred to the Web version of this article.)

Moreover, the effect of the Mg degradation, which most probably induces the observed cell proliferation inhibition was studied. Therefore, the medium pH, osmolality and Mg concentration were individually adapted to observe the influences of selected Mg degradation dependent effects (Table 5). These adapted medium conditions were used with the coculture and the cells were stained for the proliferation marker Ki-67. Fig. 7 shows the expression of Ki-67 in Saos-eGFP and RF Fibroblasts seven days after cell seeding. Relative numbers of Ki-67 positive tumor cells were significantly reduced under increased pH conditions (0.7% CO_2_). This was comparable to the results obtained for the coculture on Mg. Moreover, the increase in osmolality or just the increase in Mg concentration using the extracts did not significantly change the relative number of Ki-67 positive tumor cells compared to the control under normal cell culture conditions. But interestingly, the proportion of Ki-67 positive fibroblasts was significantly higher when incubated with extracts compared to the control. This indicates that the pH environment created by the Mg degradation may be the most compromising surface-near effect for the tumor cell proliferation.

**Table 5:** pH, osmolality and Mg concentration of the solutions that simulate selected Mg degradation-dependent effects separately.

Condition	pH	Osmolality (mOsmol/Kg)	Mg concentration (mM)	Relative number of Ki-67^+^ Saos-eGFP	Relative number of Ki-67^+^ RF Fibroblasts
glass control	7.8	335	0.8	46.7 ± 11.5	0.5 ± 1.0
Mg disc	7.9	357	4.8	8.2 ± 2.1	2.1 ± 2.7
PEG 400	7.8	412	0.8	30.7 ± 12.9	0.9 ± 2.6
0.7% CO_2_	8.5	335	0.8	3.4 ± 2.9	0.0 ± 0.0
30 mM Mg extract	7.7	359	30.0	33.8 ± 16.4	5.7 ± 9.6
5 mM Mg extract	7.7	356	5.0	36.1 ± 17.6	19.0 ± 8.5

## 4 Discussion

We used the previously described osteosarcoma-fibroblast coculture to analyze cytotoxicity and proliferation effects of slow degrading Mg and Mg–6Ag materials for the detailed investigation in this study [10]. The use of slow degrading Mg-based materials allows the analysis of cancer cell-specific cytotoxicity and the investigation of cancer characteristics during Mg material degradation. The coculture, consisting of green fluorescent bone tumor cells (Saos-eGFP) and red fluorescent healthy fibroblasts (RF Fibroblasts) was shown to be a suitable model to study influences of Mg-based materials on cancer cells. The cells were also analyzed in monocultures and on non-degrading material as controls. The Mg degradation is O_2_ dependent. The oxygen availability in the tumor tissue ranges between 0.3 and 2.1% tumor type-dependently [[11], [12], [13], [14]]. Therefore, most of the experiments were conducted under normoxia (20% O_2_) and hypoxia (3% O_2_).

With the additional use of silver (Ag)-containing Mg materials, we aimed to increase the cytotoxic potential towards the cancer cells and improve the material for a potential osteosarcoma therapy. Silver was already reported to exert antibacterial activity [37,38], which can minimize immunological reactions or sepsis as a result of an Mg-based material implantation *in vivo*. Silver nanoparticles (AgNP) were also previously reported to elevate oxidative stress, to disturb the membrane integrity, to stop the cell cycle progression, and to induce DNA damage and apoptosis in cancer cells [39]. Ag^+^, which are released from the material during Mg–6Ag degradation, will quickly bind to chloride and sulfide ions or thiol groups on proteins [40]. In this form, the entry of silver into the cell will be hindered.

Nanosilver has been previously linked to exert anticancer activity after cell entry. It dissolves intracellularly into Ag^+^, which resulted in increased reactive oxygen species (ROS) formation and DNA damages linking Ag^+^ closely to cell cycle arrests [41]. Furthermore, silver was reported to induce apoptosis in cancer cells via ROS formation, increased caspase-3 and p53 activation and lipid peroxidation [42].Initially, the influence of degrading (Mg, Mg–6Ag) and non-degrading (Ti–6Al–4V) material on the progression of tumor and healthy cells in coculture as well as in monocultures under normoxia and hypoxia was studied. Fig. 1 showed that the action of cells in coculture were totally different compared to the monocultures, indicating the relevance of using a coculture over monocultures in this study. While in the coculture healthy cell numbers steadily increased on all materials, tumor cell numbers remained relatively constant on Mg and Mg–6Ag in contrast to non-degrading Ti–6Al–4V. This may have two possible explanations:

(I) An equilibrium of tumor cell cytotoxicity and proliferation (tumor mass dormancy)
(II) A slowed/inhibited tumor cell proliferation (cellular dormancy)

To test the cytotoxicity potential of the slow degrading Mg and Mg–6Ag on the coculture, three different methods were employed and compared.

(1) The lactate dehydrogenase (LDH) release showed no significant differences between Mg-based materials and the glass control. Only the lysed control, where the LDH release was forced by permeabilization of the cells, showed significantly higher LDH values compared to Mg and Mg–6Ag. This assay is based on a color reaction from tetrazolium salt to formazan. Other tetrazolium-based cytotoxicity tests were previously shown to be interfered by Mg degradation [43].
(2) Discrimination of live and dead cells were conducted by staining primary amines and analysis with flow cytometry. Flow cytometry did not show any significant differences between cell cytotoxicity on degrading and non-degrading materials, as well. But this assay is not a direct procedure and requires a cell detachment from the material with subsequent wash steps. Therefore, the initial used cell numbers can be further reduced by washing, which will result in too little events required for flow cytometry.
(3) Caspase-3/ROS staining revealed a higher production of oxidative stress in the coculture cells on Mg and Mg–6Ag compared to the negative control under normoxia. For both, coculture and monoculture oxidative stress in the healthy cells tended to be higher than in tumor cells. In normal cells, ROS levels are balanced through multiple enzymes of the antioxidant system, e.g. super oxide dismutase [44]. In malignant cells, ROS take the role of a double-edged sword. In low concentrations, it can initiate and promote cancer development at an early stage [44]. At higher concentrations it can induce apoptotic cell death [45], or inhibit tumor cell grow as reported by Diao et al. [46]. This may be an explanation for the constant tumor cell numbers in coculture (Fig. 1): The level of ROS is high enough to reduce growth of the osteosarcoma cells, but not high enough to induce apoptosis, here represented by caspase-3 activity.

Table 3 and Fig. 2 revealed that slow degrading Mg and Mg–6Ag did not lead to tumor cell death. Furthermore, there seemed to be no differences comparing Mg and Mg–6Ag. This leads to the assumption that the potential intracellular Ag^+^ concentration was not sufficient to induce apoptosis. This may be due to the fact that Ag^+^ quickly binds other ions like chloride and sulfides and cannot easily enter the cell. Another explanation is that the released Ag from Mg–6Ag was very low. We measured Ag concentrations around 0.2 μM in the supernatant during degradation of the here used Mg–6Ag material (Table 2). In comparison to this, the literature indicated IC_50_ of different Ag sources and different cancer cells between 6.75 μM and 0.37 mM [[47], [48], [49], [50]].

Since the here used slow degrading Mg-based materials did not show a cytotoxic potential towards Saos-eGFP, the influence of Mg-based materials on cell proliferation was studied to verify the explanation (II): “inhibited tumor cell proliferation”. Both, tumor cells and healthy cells in coculture and monocultures under normoxia and hypoxia were stained for the proliferation marker Ki-67. This marker is an approved prognostic tool in clinics [51,52] and proliferation marker for *in vitro* studies [53,54].

Fig. 3 revealed that the proportion of Ki-67 positive tumor cells was overall higher compared to healthy cells. Furthermore, the relative number of Ki-67 positive, therefore proliferating cells, was significantly higher on non-degrading material compared to the here used slow degrading Mg and Mg–6Ag. This supports the findings from Fig. 1 that the overall cell numbers on non-degrading Ti–6Al–4V are higher than on the degrading materials. A possible explanation may be that the tumor cells stop proliferation and acquire a dormancy-like phenotype when the surrounding environment conditions are unfavorable. Such unfavorable conditions can be generated by the Mg degradation: elevated pH, osmolality, hydrogen evolution and Mg concentration. The cellular dormancy of the cancer cells was confirmed by the p38 expression that tended to be higher when the cancer cells were seeded on Mg and Mg–6Ag in comparison to the non-degrading glass control.

As shown in Table 4, this dormancy-like phenotype is reversible as they are able to proliferate again when the cells are detached from degrading material and are reseeded on non-degrading material. Lee and colleagues also reported reversibility when they stopped forcing breast cancer cells into dormancy [55]. The findings that Mg degradation can induce a dormancy-like state for tumor cells imply a doubled-edged sword for the application of these materials as biomedical implants in cancer therapy:

(I) Tumor cells stop proliferating, which can avoid severe/fast courses of diseases.
(II) Entering dormancy make the tumor cells inaccessible for the treatment with anti-proliferative drugs.

Therefore, in a next step, the tumor cells should be forced back into the cell cycle to resensitize them for e.g. cytostatics. PERK was previously reported to be a major inducer of cell cycle arrest upon endoplasmic reticulum (ER) stress and unfolded protein response (UPR), triggered by e.g. hypoxia [56] or oxidative stress [57], to prevent damage to the cell [[58], [59], [60]]. Fig. 5 revealed that inhibiting PERK by GSK2606414 failed to increase the relative number of Ki-67 positive cells on slow degrading Mg and Mg–6Ag materials. This suggests that the proliferation inhibition caused by the degradation of Mg and Mg–6Ag materials is induced via other signaling pathways. Inducing TGF-β signaling pathway can also lead to a cell cycle arrest by inhibition of cyclin-dependent kinases [61]. But, neither the phosphorylated SMAD2/SMAD3 complex nor IL-8 as possible downstream targets were significantly affected by the degrading material. Shi and colleagues reported increased IL-8 secretion from cancer cells under hypoxia and in an acidic environment [26]. However, we found an elevated IL-8 secretion from the coculture on Mg-based materials (Fig. 6B, hypoxia, day 3 and 7). This may be due to the lower cell ratio of Saos-eGFP:RF Fibroblasts on degrading Mg and Mg–6Ag compared to the non-degrading controls. Indeed, Mussano et al. showed very low IL-8 expression in Saos-2 [62], from which Saos-eGFP originated.

Several authors explained further possible strategies to directly target dormant cancer cells [63,64]. One approach is to keep the dormant cancer cells in their dormant state. This may be the approach of a continuous degradation of Mg implants. It can inhibit cancer recurrence and metastases; however, this requires a continuous therapy to avoid a reawakening of the cancer cells. A second approach aims to awake the dormant cancer cells and sensitize them to antiproliferative drug therapy. This may lead to more aggressive phenotypes of the cancer. The last approach is the eradication of dormant cancer cells. This may be the most suitable therapy approach with regard to Mg-based material implants. The degrading Mg material specifically forces the cancer cells into a dormant state. A drug combined with the Mg material may then target these dormant cells, for example IGF-1R inhibitors [63,64].

The present study showed that the here used slow degrading Mg-based materials specifically reduced tumor cell proliferation in a coculture model under normoxia and hypoxia. However, Mg degradation is a very complex mechanism inducing increased pH and osmolality as well as a constant Mg ion and hydrogen gas release at the Mg surface. We investigated the influence of selected surface-near effects of Mg degradation on the cell proliferation phenotype of the coculture. Although global pH and osmolality values for the degradation of these materials were previously published [10], higher values were assumed at the direct surface as suggested by Lamaka et al. [65]. Normally, solid tumors acidify their microenvironment by an excessive metabolism (Warburg effect) and acid extrusion [66], whereby this acidification can even surpass global alkalization by Mg degradation [67]. In that way, these tumor cells can maintain a proliferative and malignant phenotype [[68], [69], [70]]. Furthermore, other studies already showed that a neutralization/alkalization of the acidic extracellular pH can reduce overall tumor growth [[71], [72], [73], [74]]. These results are also consistent with our findings regarding the proliferative fraction of tumor cells (Fig. 7). While increasing the osmolality and Mg^2+^ concentration did not lead to a significant change of the relative number of proliferating tumor cells, alkalizing the pH reduced the Ki-67 positive tumor cells similar to the Mg degradation. Interestingly, an elevation of Mg concentration increased the relative number of proliferating healthy fibroblasts. This may explain the increasing fibroblast numbers in coculture observed on the Mg and Mg–6Ag materials (Fig. 1). Measuring and manipulating the hydrogen gas release as another event of Mg degradation will be part of future studies. A previous study of Zan et al. showed decrease tumor growth *in vitro* and *in vivo* using implantable Mg wires [17]. The authors interpreted that this growth inhibition resulted from released H_2_ but did not consider other surface-near effects. They further demonstrated that a concentration of around 60 μM of dissolved H_2_ was sufficient to induce apoptosis in the tumor cells, while a lower concentration rarely affected the cell proliferation and viability. This means that the effect of H_2_ is less remarkable for slow degrading Mg materials, in the context of our results.

Taken together, the local pH around the Mg surface is the most probable Mg degradation dependent surface-near effect that is responsible for the observed tumor cell proliferation inhibition by slow degrading Mg-based materials. In contrast to that, increasing the Mg^2+^ concentration and associated osmolality as in Mg extracts did not lead to the same results as obtained directly on Mg. This underlines the importance of a direct model on Mg-based materials over the usage of Mg extracts to obtain reliant results.

## 5 Conclusion

Our results showed that Mg-based materials are suitable to use in osteosarcoma therapy. Although, the slow degrading materials did not induce tumor cytotoxicity, the here used Mg and Mg–6Ag significantly reduced the cancer cell proliferation. Therefore, Mg-based materials may be used in combination with other drugs to directly target dormant cancer cells [75]. The Mg degradation dependent pH increase was demonstrated to be the driving factor of forcing cancer cells into dormancy. Aside from this, the neutralization of the pH around the cancer cells can be beneficial for drug entry of weak basic chemotherapeutics [76]. In this way, Mg-based materials can increase chemotherapeutic efficacy, therefore reduce the dose of the chemotherapeutic agent and can prevent drug resistances. Hence, the central importance of Mg-degradation dependent pH increase should be considered for future material designs.

## Funding

This work was funded by the Helmholtz – 10.13039/501100006769Russian Science Foundation Joint Research Groups HRSF-0025.

## CRediT authorship contribution statement

**Philipp Globig:** Conceptualization, Formal analysis, Investigation, Methodology, Validation, Visualization, Writing – original draft, Writing – review & editing. **Regine Willumeit-Römer:** Conceptualization, Funding acquisition, Project administration, Supervision, Writing – review & editing. **Fernanda Martini:** Resources. **Elisa Mazzoni:** Resources. **Bérengère J.C. Luthringer-Feyerabend:** Conceptualization, Methodology, Project administration, Supervision, Writing – review & editing.

## Declaration of competing interest

None.
